# Associations between parental chronic pain and self-esteem, social competence, and family cohesion in adolescent girls and boys – family linkage data from the HUNT study

**DOI:** 10.1186/s12889-015-2164-9

**Published:** 2015-08-22

**Authors:** Jannike Kaasbøll, Ingunn Ranøyen, Wendy Nilsen, Stian Lydersen, Marit S. Indredavik

**Affiliations:** Regional Centre for Child and Youth Mental Health and Child Welfare (RKBU) of Central Norway, Faculty of Medicine, Norwegian University of Science and Technology (NTNU), Postbox 8905, Medisinsk teknisk forskningssenter (MTFS), N-7491 Trondheim, Norway; Department of Child Development and Mental Health, Division of Mental Health, Norwegian Institute of Public Health, PO Box 4404, Nydalen, 0403 Oslo, Norway; Department of Child and Adolescent Psychiatry, St. Olav’s Hospital, Trondheim University Hospital, Postbox 6810, Elgeseter, N-7433 Trondheim, Norway

## Abstract

**Background:**

Parental chronic pain has been associated with adverse outcomes in offspring. However, knowledge on individual and family resilience factors in adolescent offspring of chronic pain sufferers is scarce. This study thus aimed to investigate the associations between parental chronic pain and self-esteem, social competence, and family cohesion levels reported by adolescent girls and boys.

**Methods:**

Based on cross-sectional surveys from the Nord Trøndelag Health Study (the HUNT 3 study), the study used independent self-reports from adolescents aged 13 to 18 years (*n* = 3227) and their parents and conducted separate linear regression analyses for girls and boys.

**Results:**

Concurrent maternal and paternal chronic pain was associated with reduced self-esteem, social competence, and family cohesion in girls. Moreover, maternal chronic pain was associated with higher social competence in boys and reduced self-esteem in girls. The majority of the observed associations were significantly different between girls and boys. Paternal chronic pain was not found to be associated with child outcomes.

**Conclusions:**

The findings indicate that the presence of both maternal and paternal chronic pain could be a potential risk factor for lower levels of individual and family resilience factors reported by girls. Further research on the relationship between parental pain and sex-specific offspring characteristics, including positive resilience factors, is warranted. The study demonstrates the importance of targeting the entire family in chronic pain care.

## Background

Parents with chronic pain constitute a significant proportion of the adult population in Europe, as approximately 20 % of adults live with moderate to severe chronic pain [1]. Research suggests that chronic pain has a substantial impact on a family’s general well-being [2–4], and according to recent studies, parental chronic pain and physical illness are associated with children’s physical health [5, 6] and psychological adjustment [7–9]. We have previously demonstrated associations between concurrent maternal and paternal chronic pain and symptoms of anxiety and depression in girls and boys, as well as smoking and alcohol intoxication in boys [9, 10]. In line with these findings, Pedersen and Revenson [11] argue that research on parental illness and child outcomes should include more positive and growth-related outcomes in addition to traditional indices of psychopathology.

Resilience is defined in terms of factors that contribute to good psychological outcomes despite the presence of risk factors [12]. Individual characteristics such as high self-esteem and high social competence and familial characteristics such as family cohesion are considered resilience factors [13], and high levels of these factors are considered crucial for children’s ability to cope with life stressors [14–18]. *Self-esteem*, which refers to positive or negative self-evaluations [19], has been reported to protect against the damaging effects of a wide variety of risk factors, such as stress and depression [15, 20]. *Social competence*, which refers to one’s ability to get along with other people and to form close relationships [21], is a fundamental aspect of children’s psychological adjustment [22]. *Family cohesion*, which refers to the emotional connections between family members, is central to families’ overall functioning and children’s psychological adjustment [17, 23]. Possible associations between parental chronic pain and resilience factors are less well established, however.

The development of individual and family resilience factors involves complex mechanisms. Self-esteem, social competence, and family cohesion may be influenced by hereditary factors, observational learning, and the dynamics of family interactions [24–27]. However, parental chronic pain may directly and indirectly affect the quality of family relationships [28] by increasing the caregiver’s burden on the offspring and by decreasing the caregiver’s emotional availability, which is a risk factor for dysfunctional parenting. These factors are all associated with lower levels of self-esteem and social competence in offspring [22, 29]. Thus, research is needed on resilience factors in families that are affected by chronic pain.

Resilience factors may differ between girls and boys. Reported self-esteem in adolescence is generally higher among boys than among girls, and self-esteem decreases with age in both adolescent girls and boys [30–32]. Independent of age, girls report having greater social sensitivity, emotional regulation, and social skills than do boys [33, 34]. However, some studies have found higher self-reported levels of social competence in adolescent boys than in adolescent girls [35–37]. Moreover, in a population-based study, boys reported slightly higher perceived family cohesion than did girls [35]. Given these general findings, the associations between parental chronic pain and resilience factors in children may depend on sex and age.

Previous findings on the association between parental chronic pain and resilience factors in the offspring are somewhat ambiguous. Hirsch et al. [38] reported that children (12 to 18 years of age) of a parent with arthritis had lower self-esteem than children of a parent without arthritis. Furthermore, Chun et al. [39] found that children (6 to 16 years of age) whose fathers had chronic pain were rated by their parents as less socially competent than children of female patients, and Evans et al. [25] found that mothers with chronic pain reported lower social functioning for their male offspring (6 to 12 years of age) than did mothers without parental chronic pain. Dura and Beck [40] reported higher degrees of conflict in families experiencing chronic pain than in other families, whereas the findings from Smith and Chambers [41] suggest that children (8 to 15 years of age) of parents with recurrent headaches might not be at risk for poor psychological and family functioning. In addition, chronic pain is associated with lower socioeconomic status, which could be a potential risk factor for child adjustment [7, 18, 27, 42–44].

Most studies on parental chronic pain are limited by including only one parent, mainly the mother as the primary caregiver, whereas the effects of chronic pain among fathers have been studied less frequently [28, 45]. In addition, the effects of having two parents with chronic pain have been reported to be greater than the effects of having one or no parent with chronic pain [6, 9, 10]. Hence, research on this topic should include independent information on the offspring’s parents. Furthermore, most of the aforementioned studies are limited by their use of small clinical samples, which reduces the generalizability of the reported results, and their use of only one informant, which increases the likelihood of shared-method bias. To our knowledge, no previous study has investigated resilience factors in adolescent children of parents with chronic pain in a population-based sample by using independent reports from the mother, father, and child.

The present study aimed to explore associations between parental chronic pain and individual (self-esteem and social competence) and family (family cohesion) resilience factors in the adolescent offspring. Specifically, we aimed to examine sex-specific associations for adolescents whose parents (mother, father, or both) did or did not have chronic pain while adjusting for confounders such as socioeconomic factors and age. We hypothesized that children of parents with chronic pain would report reduced self-esteem, social competence, and family cohesion compared with children for whom neither parent had chronic pain. We further hypothesized that girls would report lower levels of self-esteem and also possibly lower levels of social competence and family cohesion, compared with boys.

## Methods

### Study design and procedures

The current study used data from a large population-based survey conducted between 2006 and 2008, the Nord-Trøndelag Health Study (the HUNT 3 study), which included the entire adolescent and adult population aged 13 years and older in the county of Nord-Trøndelag of central Norway. In the adolescent part of the survey (the Young-HUNT 3 study), the target group was 13 to 19 years old. The adolescents completed a comprehensive, self-administered questionnaire about their health and lifestyles during a school lesson. Participants aged 20 or older were invited to participate in the adult part of the survey (the HUNT 3 study). The parental and adolescent data were merged through the Norwegian family register by using the 11-digit personal number by which every citizen in Norway is registered. Data from these sources are used extensively in international research and have been the basis for a large number of peer-reviewed papers and doctoral-theses. The HUNT 3 study is described in more detail in previous publications [46, 47], and an English translation of the questionnaire is available at http://www.ntnu.edu/hunt/data/que.

### Participants

In the HUNT 3 study, there were 8200 adolescents (78 % participation rate) and 50,839 adults (54 % participation rate). The present sample comprised adolescents in the Young-HUNT 3 study for whom *both* parents participated in the adult HUNT study (*n* = 3436). A small number of participants were 12 years old (*n* = 19), 19 years old (*n* = 157) or 20 years old (*n* = 33), and, because of their limited participation, they were excluded from the analyses. Thus, the sample for the present study comprised 3227 adolescents aged 13–18 years. The adolescents were divided into four groups according to their parental chronic pain statuses: 608 (20 %) had mothers with chronic pain (M-group), 495 (16 %) had fathers with chronic pain (F-group), and 230 (7 %) had both parents with chronic pain (MF-group). The reference group comprised 1740 (57 %) adolescents who had no parents with chronic pain. In all, 154 adolescents had missing data for the items on parental chronic pain. The mean age was 15.7 (SD 1.67) years for girls (*n* = 1594) and 15.8 (1.62) years for boys (*n* = 1633). The number of girls and boys in each group by parental chronic pain status is presented in Table 1. In the present sample, 74 % of the girls and 69 % of the boys were living with both biological parents (cohabitation status is described in detail in Kaasbøll et al. [9]). Of these adolescents, 538 (33 %) had one sibling in the study (four had step-siblings) and 40 (4 %) had two siblings in the study, while 2031 (63 %) did not have any participating sibling in the study. The number of adopted adolescents was 16. Adult nonparticipants had lower socioeconomic status, were more often men, and had more mental distress and poorer health than adult participants [47]. Nonparticipating adolescents were more often older and male than participating adolescents, and if they were enrolled in school, they more often attended vocational classes than academic classes [46].

**Table 1:** Ages and education levels of parents with and without chronic pain

	Mothers				Fathers			
	Chronic pain		No chronic pain		Chronic pain		No chronic pain	
	*n* = 838		*n* = 2230		*n* = 725		*n* = 2245	
	*n* ^a^	Mean (SD)	*n*	Mean (SD)	*n*	Mean (SD)	*n*	Mean (SD)
Parental age (years)	836	44.64 (5.01)	2230	44.62 (4.98)	722	48.17 (6.08)	2345	47.48 (5.56)
	*n*	(%)	*n*	(%)	*n*	(%)	*n*	(%)
Education^b^								
Compulsory	138	(16.5)	200	(9.0)	126	(17.4)	267	(11.4)
Upper secondary	436	(52.0)	1009	(45.4)	453	(62.7)	1372	(58.5)
Higher level (tertiary)	264	(31.5)	1014	(45.6)	144	(19.9)	705	(30.1)
	838	(100.0)	2223	(100.0)	723	(100.0)	2344	(100.0)

**Note**

^a^All *n* are reported for complete cases, and the results are based on MI analyses

^b^Compulsory (<10 years), Upper secondary (10–12 years), and Higher level (tertiary) education (≥13 years)

### Measures

#### Parental variables

Parental chronic pain was measured by combining the item “Do you have physical pain now that has lasted more than 6 months?” (“No/”Yes”) with a pain-rating scale including 6 response categories for the item “How strong has your physical pain been during the last four weeks?” Responses for this item ranged from no pain (0) to very strong pain (5). This verbal rating scale is commonly used and is recommended as a global measurement of pain severity [48]. A cut-off point was set at the midpoint of the scale (no, very mild, mild, moderate, severe, very severe) because this cut-off has been useful for identifying people with clinically significant pain [49]. Hence, the assessment of parental chronic pain was based on the combination of experiencing pain lasting more than six months and experiencing moderate, severe, or very severe pain during the past month.

Socioeconomic status was measured by the highest level of education attained for both parents, which was obtained from the National Education Database (NUDB). For the current analyses, data from 2008 were used. Educational attainment was classified into the following three levels: compulsory education (<10 years), upper secondary education (10–12 years), and higher-level (tertiary) education (≥13 years).

#### Adolescent variables

Self-esteem was measured by a short version of the Rosenberg Self-Esteem Scale [50], which consisted of the following four statements: “I have a positive attitude toward myself”, “ I feel rather useless at times”, “I feel that I don’t have much to be proud of”, and “I feel that I am a valuable person, at least equal to other people”. The responses ranged from “I totally agree” (1) to “I totally disagree” (5)*.* Scores were inversed for the first and last items, and as such, higher scores indicate higher levels of self-esteem. In the present study, Cronbach’s α for the self-esteem scale was 0.79 for girls and 0.68 for boys.

Social competence and family cohesion were measured with four items each from shortened subscales of the Resilience Scale for Adolescents (READ) [51]. The scale for social competence consists of the following items: “I make others feel comfortable around me”, “I easily find new friends”, “I am good at talking to new people”, and “I always find something fun to talk about”. The scale for family cohesion consists of the following items: “In my family, we share views of what is important in life”, “I feel comfortable with my family”, “My family view the future as positive, even when sad things happen”, and “In my family, we support each other”. The responses ranged from totally disagree (1) to totally agree (5). The READ has been shown to possess adequate psychometric properties [36, 52–55]. In the present study, Cronbach’s α was 0.83 for girls and 0.81 for boys for the social competence scale and 0.87 for girls and 0.82 for boys for the family cohesion scale.

### Statistical analyses

Cronbach’s α was used to quantify the reliability and internal consistency of the scales. For all scales, mean score indices were computed if at least 50 % of the items were answered. Pearson’s chi-squared test was used to compare education levels between parents with and parents without chronic pain, and independent sample t-tests were used to compare parental age differences between the groups and differences between girls and boys within the different parental chronic pain groups. *Cohen’s d* was calculated as a function of the means and SDs of the outcome scores. As a rule of thumb, effect sizes of *d* = .30, *d* = .50, and *d* = .80 can be considered small, medium and large, respectively [56]. Multiple linear regression analyses were used to examine the associations between parental chronic pain and adolescents’ levels of self-esteem, social competence and family cohesion. Parental chronic pain was used as a categorical covariate with four categories and the dependent variables were self-esteem, social competence, and family cohesion, respectively. Potential confounding factors were identified based on a priori knowledge [57] and included child age and parental education. Parental education was coded: 1 = compulsory, 2 = upper secondary, 3 = higher level (tertiary), and used as a covariate. These covariates were adjusted for simultaneously in the next step of the analyses.

Multiple imputation (MI) was used to reduce bias and to avoid a loss of sample size caused by missing data. We imputed m = 100 data sets, as recommended by Buuren [58]. The original data (without MI) were used to describe (cross-tabs) parental education. All regression analyses were based on MI. The analyses with and without imputed data provided similar results. To test sex differences in the associations between parental chronic pain and resilience factors in offspring, we combined the imputed data sets for girls and boys as suggested by van Buuren [58], and we included interaction terms between parental chronic pain and child sex. In all other analyses were conducted separately for the imputed data sets for boys and girls. Only results from the imputed data are presented. Where relevant, 95 % CIs are reported. Two-sided *p*-values < 0.05 were considered significant. SPSS version 21.0 was used for the data analyses.

### Ethics

Written informed consent to participate in the HUNT study was provided by all participants and by the parents of the children who were under the age of 16 years. The present study complied with the Declaration of Helsinki principles, and it was approved by the Regional Committee for Medical Research Ethics (REK; reference number 4.2008.664) and by the Norwegian Social Science Data Services (NSD).

## Results

### Descriptive statistics

The prevalence of chronic pain was 27 % for mothers (*n* = 838) and 24 % for fathers (*n* = 725). Parents with chronic pain had lower education levels compared with parents without chronic pain (*p* < .001). Furthermore, fathers with chronic pain were slightly older than fathers without chronic pain (*p* = .004) (Table 1). The mean values for self-esteem, social competence, and family cohesion for girls and boys are presented in Table 2 by parental chronic pain status.

**Table 2:** Self-esteem, social competence, and family cohesion by parental chronic pain status

	Maternal chronic pain			Paternal chronic pain			Concurrent maternal and paternal chronic pain			Reference^d^		
	*n* ^a^	Mean	SE	*n*	Mean	SE	*n*	Mean	SE	*n*	Mean	SE
Self-esteem^b^												
Girls (*n* = 1594)	290	2.86	0.04	255	2.91	0.04	106	2.81	0.06	857	2.97	0.02
Boys (*n* = 1633)	303	3.26	0.03	226	3.23	0.04	122	3.21	0.05	853	3.24	0.02
Social competence^c^												
Girls (*n* = 1594)	291	3.72	0.05	255	3.76	0.05	106	3.56	0.08	851	3.79	0.03
Boys (*n* = 1633)	302	4.02	0.04	222	3.90	0.05	120	4.03	0.07	840	3.91	0.03
Family cohesion^c^												
Girls (*n* = 1594)	291	4.18	0.05	255	4.15	0.05	106	4.03	0.08	852	4.25	0.03
Boys (*n* = 1633)	302	4.32	0.04	223	4.35	0.05	120	4.27	0.06	840	4.30	0.02

^a^All *n* are reported for complete cases, and the results are based on MI analyses. ^b^Rosenberg Self-Esteem scale, range from 1 to 4, where a high score indicates high self-esteem ^c^Resilience Scale for Adolescents (READ), range from 1 to 5, where a high score indicates high social competence and family cohesion

### Individual and family factors – sex differences within the parental chronic pain gruop

Self-esteem scores were significantly lower for girls than for boys in all parental chronic pain groups: maternal chronic pain group (M-group), t(542) = −8.47, *p* < .001 (Cohen’s d = −0.73); paternal chronic pain group (F-group), t(479) = −6.24, *p* = .048 (Cohen’s d = −0.18); concurrent maternal and paternal chronic pain group (MF-group), t(226) = −4.74, *p* < .001 (Cohen’s d = −0.59); and no parental chronic pain group (reference group), t(1780) = −10.12, *p* < .001 (Cohen’s d = −0.48).

Social competence scores were significantly lower for girls than for boys in the M-group, t(568) = −4.95, *p* < .001 (Cohen’s d = −0.41); the MF-group, t(198) = −3.92, *p* < .001 (Cohen’s d = −0.56); and the reference group, t(1689) = −3.25, *p* = .001 (Cohen’s d = −0.16). The results showed a tendency toward lower social competence for girls compared with boys in the F-group, t(475) = −1.95, *p* = .051(Cohen’s d = −0.18).

Family cohesion scores were significantly lower for girls than for boys in the M-group, t(568) = −2.12, *p* = .034 (Cohen’s d = −0.18); the F-group, t(475) = −2.93, *p* = .003 (Cohen’s d = −0.26); and the MF-group, t(224) = −2.11, *p* = .036 (Cohen’s d = −0.28). There were no significant differences between girls and boys in the reference group, t(1690) = −1.29, *p* = .198 (Cohen’s d = −0.06).

### Associations between parental chronic pain and individual and family resilience factors

For girls only, maternal chronic pain (b = −0.12, CI: −0.19 to −0.04, *p* = .003) and concurrent maternal and paternal chronic pain (b = −0.17, CI: −0.29 to −0.06, *p* = .003) were associated with lower *self-esteem*. These associations were slightly attenuated when the analysis was adjusted for child age and parental education (Table 3). Interaction analyses confirmed that the association between maternal pain and offspring self-esteem was present for girls but not for boys (*p* = .016). However, the interaction between concurrent maternal and paternal chronic pain and child sex was not significant (*p* = .086). Paternal chronic pain was not significantly associated with self-esteem for girls or boys (Table 3).

**Table 3:** Associations between parental chronic pain and self-esteem, social competence, and family cohesion in adolescents Linear regression

		Maternal chronic pain		Paternal chronic pain		Concurrent maternal and paternal chronic pain	
	*n* ^a^	b 95 % CI	*p*	b 95 % CI	*p*	b 95 % CI	*p*
Self-esteem^b^							
*Girls*							
Unadjusted	1508	−0.12 [−0.19, −0.04]	.003	−0.06 [−0.14, 0.02]	.127	−0.17 [−0.29, −0.06]	.003
Adjusted^c^	1501	−0.10 [−0.17, −0.02]	.014	−0.04 [−0.12, 0.04]	.279	−0.14 [−0.26, −0.03]	.016
*Boys*							
Unadjusted	1504	0.02 [−0.05, 0.09]	.605	−0.02 [−0.10, 0.06]	.627	−0.04 [−0.14, 0.07]	.512
Adjusted	1495	0.03 [−0.04, 0.11]	.378	−0.01 [−0.09, 0.07]	.803	−0.01 [−0.11, 0.10]	.930
Parental chronic pain × child sex^c^			.016		.564		.086
Social competence^d^							
*Girls*							
Unadjusted	1492	−0.07 [−0.17, 0.04]	.203	−0.03 [−0.14, 0.08]	.619	−0.24 [−0.40, −0.08]	.003
Adjusted	1489	−0.07 [−0.17, 0.04]	.213	−0.02 [−0.13, 0.09]	.687	−0.24 [−0.40, −0.08]	.004
*Boys*							
Unadjusted	1484	0.12 [0.02, 0.22]	.019	−0.01 [−0.13, 0.10]	.858	0.13 [−0.01, 0.28]	.076
Adjusted	1475	0.12 [0.02, 0.22]	.018	−0.01 [−0.13, 0.10]	.849	0.14 [−0.01, 0.29]	.070
Parental chronic pain × child sex^c^			.011		.888		.001
Family cohesion^d^							
*Girls*							
Unadjusted	1504	−0.08 [−0.18, 0.02]	.130	−0.11 [−0.22, −0.00]	.042	−0.24 [−0.40, −0.09]	.002
Adjusted	1497	−0.06 [−0.17, 0.04]	.232	−0.10 [−0.20, 0.01]	.081	−0.22 [−0.37, −0.06]	.007
*Boys*							
Unadjusted	1485	0.02 [−0.08, 0.12]	.683	0.05 [−0.06, 0.16]	.340	−0.02 [−0.17, 0.12]	.751
Adjusted	1497	0.03 [−0.07, 0.13]	.515	0.06 [−0.05, 0.17]	.304	0.01 [−0.15, 0.15]	.938
Parental chronic pain × child sex^c^			.189		.149		.041

^a^All *n* are reported for complete cases, and the results are based on MI ^b^Rosenberg Self-Esteem Scale, range from 1 to 4, where a high score indicates high self-esteem ^c^Adjusted for child age and maternal and paternal education ^d^Resilience Scale for Adolescents (READ), range from 1 to 5, where a high score indicates high social competence and family cohesion

Concurrent maternal and paternal chronic pain was significantly associated with lower *social competence* for girls (b = −0.24, CI: −0.40 to −0.08, *p* = .003), but boys in the MF-group showed a tendency toward higher social competence (b = 0.13, CI = −0.01 to 0.28, *p* = .076). The associations withstood adjustments for child age and parental education, and these observed sex differences were statistically significant in the interaction analysis (*p* = .001). No significant associations were found between maternal (*p* = .203) or paternal chronic pain (*p* = .619) and social competence for girls (Table 3). For boys, there was a significant association between maternal chronic pain and increased social competence (b = 0.12, CI: 0.02 to 0.22, *p* = .019). Adjusting for age and parental education did not influence the association, and interaction analyses confirmed this sex difference (*p* = .016).

Concurrent maternal and paternal chronic pain was significantly associated with lower *family cohesion* for girls only (b = −0.24, CI: −0.40 to −0.09, *p* = .002). This association remained after the analysis was adjusted for child age and parental education, and this sex difference was statistically significant (*p* = .041). The association between paternal chronic pain and reduced family cohesion reported by girls (b = −0.11, CI: −0.22 to −0.00, *p* = .042) became nonsignificant in the adjusted analysis (b = −0.10, CI: −0.20 to 0.01, *p* = .081). Maternal chronic pain was not significantly associated with reported family cohesion for either girls or boys (Table 3).

## Discussion

In the present study, we explored the association between parental chronic pain and individual and family resilience factors reported by adolescent girls and boys. Three interesting findings are worth highlighting: concurrent maternal and paternal chronic pain was associated with reduced self-esteem, social competence, and family cohesion in girls, maternal chronic pain was associated with higher social competence in boys, and paternal chronic pain was not associated with any child outcomes.

First, the most prominent finding was that although chronic pain in both parents was associated with the expected reductions in self-esteem, social competence, and family cohesion in girls, parental chronic pain was not associated with negative outcomes for boys. In addition, girls had reduced self-esteem when only their mother had chronic pain. The majority of the observed associations were found to be significantly different between girls and boys. Parental chronic pain has been linked to increased levels of familial stress and to parent–child conflict [2]. In the current study, girls reported lower levels of family cohesion than did boys in all parental chronic pain groups, whereas no sex difference was found in the reference group. Thus, girls may be more sensitive to strain in the family than boys when one or both parents have chronic pain, which may influence girls’ perceptions of family cohesion.

Another interpretation of the sex differences could be linked to the caregiving roles that can arise in families of individuals with parental chronic pain [2], whereby parents may withdraw from their caregiving responsibilities [3, 45]. Previous studies have indicated that girls may be more likely than boys to take on care responsibilities in the home when their parents are ill, and other studies have reported that the burden of being a young caregiver may be heavier for girls than for boys [59–61]. Such caregiving roles and responsibilities are likely to occur when both parents suffer from chronic pain. Moreover, sex differences in caregiving roles and responsibilities in the home may increase the effect of conditioning and modeling processes arising from parental pain-related behavior on girls.

In the present study, girls reported lower levels of personal and familial resilience factors than did boys in most of the groups, with moderate effect sizes. This finding is consistent with those of previous studies [30, 35, 36]. When girls reach adolescence, they become more vulnerable to stressors, especially interpersonal stress [62, 63]. Further, during adolescence, family interactions change to meet the adolescents’ developmental needs for individuation and independence [22]. Friends rather than parents become increasingly important [34, 64]. One may thus speculate that a combination of girls’ increased focus on relationships and their high caregiver burden, especially when both parents have chronic pain, may contribute to their concerns about social approval, abandonment, and the status of their friendships, which may influence their well-being [65]. Further research could benefit from investigating sex differences in stress responses and coping related to parental illness and the possible impact of caregiving roles.

Second, the results of the current study indicate that maternal chronic pain was, somewhat surprisingly, associated with higher levels of self-reported social competence in boys. In addition, boys whose mothers and fathers both had chronic pain showed a trend toward greater social competence. These results are in contrast to those of previous studies reporting that maternal and paternal chronic pain was associated with decreased social competence in boys [39, 45]. However, these studies investigated younger children in small clinical samples and used parental reports. As an explanation of the finding in the present study, maternal chronic pain may provide opportunities for positive development in boys. Indeed, Evans et al. [66] suggested that maternal chronic pain may enhance growth in addition to adversity in many families. One might also speculate that in the presence of parental chronic pain, boys may seek an escape from the family context, looking for social support and activities outside the family, which could influence their perceptions of their own social competence.

Third, in the present study, paternal pain was not associated with any of the adolescent resilience factors. This finding conflicts somewhat with the results of Chun et al. [39], who found that children (6 to 16 years old) whose fathers had chronic pain were rated by their parents as less socially competent than children whose fathers did not have chronic pain. However, Evans et al. [25] found that paternal chronic pain was not associated with child (6 to 12 years old) outcomes and argued that in the context of pain, mothers may have a stronger influence than fathers. The discrepancies in these results could be attributable to the aforementioned differences in study design. Despite societal changes in the industrialized countries that have more women in the workforce, mothers are still more likely than fathers to take on child care responsibilities [67]. Further, the literature indicates that women display more pain-related behavior compared with men [68]. Consequently, adolescents may witness their mothers’ suffering more frequently than their fathers’ suffering.

### Limitations and strengths

A major limitation of the present study was the cross-sectional design. Demonstrating temporal precedence was not possible because all variables were measured simultaneously. The results should be confirmed in future prospective studies, preferably by using longitudinal data with multiple study waves [69, 70]. However, the present results can serve as a basis for constructs that warrant further investigation for a broader understanding of the mechanisms at work in families of chronic pain sufferers. The current study was based solely on self-reports. Future studies could thus benefit from including observational or multi-informant designs. Another limitation is that adolescents who did not have both parents participating in the HUNT 3 study were not included in the main analysis. However, the effect sizes of the differences between the groups were previously reported to be small [71]. Some of the null findings in the current study nevertheless must be interpreted with care because of the attrition rates. Furthermore, our analyses did not control for statistical dependency between siblings. This may cause the computed CIs to be too narrow. However, the percentage of siblings in our study was relatively low, and between subject correlations are usually low, so the computed CIs can be regarded as good approximations. Generally, Cronbach’s α values at 0.70 or higher are considered to indicate high reliability. However, the Cronbach’s α for self-esteem scale among boys was 0.68.

The present study has a number of strengths, such as the use of a large population sample and the inclusion of independent information on children and both parents. The study could thus examine the unique contributions of maternal, paternal, and concurrent maternal and paternal chronic pain by comparing families experiencing such chronic pain with families without parental chronic pain. The large sample size also rendered an investigation of the effects of having one or both parents with chronic pain possible. Moreover, the large sample size allowed for an approach to examining sex differences in which the effects of both parental and offspring sex could be assessed.

### Clinical and scientific implications

The present study expands the existing literature and supplements today’s knowledge on the relationship between parental chronic pain and child outcomes. Knowledge about the resilience factors displayed by children of chronic pain sufferers is important, as the family environment is central in chronic pain treatment [2]. Despite the cross-sectional study design, our findings indicate that a possible direction for future work may be to explore intervention strategies that are designed to increase self-esteem and social competence, especially among girls who have two parents with chronic pain. According to recent studies on developmental cascades and trajectories of social competence and behavioral adjustment [72, 73, 16, 74, 21], social competence is an antecedent of behavioral adjustment, including the internalization of problems such as anxiety and depression. Thus, for parents and health care practitioners, promoting children’s social competence may be beneficial and may protect children against later symptoms of anxiety and depression [72, 23]. Further research should aim to investigate protective factors for adverse outcomes in offspring when parents are suffering from chronic pain. Furthermore, resiliency in individual members is important to family resiliency. Hence, individual and family resiliency in individuals and family members of chronic pain sufferers should also be investigated [75, 76]. It would also be a contribution to the current literature to investigate genetic and environmental effects of parental chronic pain.

## Conclusions

Using data from a large population-based study, the present study addresses gaps in the existent literature by examining links between parental chronic pain and individual and family resilience factors reported by adolescent girls and boys. The most prominent finding was that concurrent maternal and paternal chronic pain was associated with reduced self-esteem, social competence, and family cohesion in girls. In addition, maternal chronic pain was associated with high social competence in boys and low self-esteem in girls, whereas paternal chronic pain was not associated with any child outcomes. Further knowledge about the role of potential protective factors in relation to adverse outcomes would be beneficial for understanding the full range of children’s experiences with parental chronic pain.
